# The Story of the Insane from Year to Year

**Published:** 1896-02-29

**Authors:** 


### THE STORY OF THE INSANE FROM YEAR TO YEAR.

EIGHTH SERIES.

### CUMBERLAND AND WESTMORELAND ASYLUM.

On January 1st this asylum contained 579 patients, and at the end of December there were 580. There were 173 admissions, 83 recoveries, and 58 deaths. The recoveries stand at 47·9 per cent. of the admissions and the deaths at 9·9 on the average number resident. Fourteen of the deaths were from cerebral and spinal diseases, and 12 from consumption. Intemperance in drink caused the insanity in 17 instances, and hereditary tendency in 46, so that nearly 50 per cent. of the year's admissions were due to preventable causes. Dr. Campbell, when referring to his high recovery rate, says, "If the average recovery rate in English County and Borough Asylums had been for 22 years anything approaching what it has been here, we should not hear so much about the increase of insanity by accumulation as we do." Perhaps not; but the amount of insanity in the next generation would be increased, and Dr. Campbell should reflect that his high recovery rate is not all gain. There are about 40 private patients in this asylum, and separate accommodation is being provided for them. Every medical superintendent can in his heart echo what Dr. Campbell says about the cares and responsibilities of office; and "when a year passes without any extremely grave accident or combination of evils, it is a matter of thankfulness to those in immediate charge, knowing as they do how easily it might have been otherwise." The weekly rate of maintenance is 9s. per head.

